# Sexual health knowledge, sources of information, and associated attitudinal disconnects among university students

**DOI:** 10.1590/1806-9282.20260407

**Published:** 2026-08-07

**Authors:** Abdulsamet Bağcı, Betül Battaloğlu İnanç

**Affiliations:** 1 Ministry of Health, Ordu State Hospital – Ordu, Türkiye.; 2 Muğla Sıtkı Koçman University, Faculty of Medicine, Department of Family Medicine – Muğla, Türkiye.

**Keywords:** Health literacy, Sexual behavior, Students, Health services accessibility, Health knowledge, attitudes, practice

## Abstract

**OBJECTIVE:**

The aim of this study was to evaluate university students’ knowledge, attitudes, and behaviors regarding sexual and reproductive health.

**METHODS:**

This cross-sectional study was conducted using face-to-face questionnaires administered on a voluntary basis to university students aged 18-26 presenting to the Muğla Sıtkı Koçman University Kötekli No. 4 Family Health Center. Data were analyzed using International Business Machines Statistical Package for the Social Sciences Statistics version 26.0. Associations between categorical variables were evaluated using the chi-square and Fisher’s exact tests.

**RESULTS:**

A total of 383 students participated in the study (55.4% female, n=212; 44.6% male, n=171). Of the participants, 42.3% reported having sexual experience, which was significantly higher among males (p=0.002). The most common source of information on sexual and reproductive health was the internet (65.3%). The best-known family planning method was the condom (98.7%). The most commonly known sexually transmitted infection was human ımmunodeficiency virus/acquired ımmunodeficiency syndrome, and the most frequently reported symptom was genital discharge.

**CONCLUSION:**

University students’ level of knowledge regarding sexual and reproductive health is insufficient, and this gap suggests that knowledge alone is insufficient to drive behavior change, and that sociocultural and cognitive barriers—such as stigma, perceived norms, and risk misperception—play a mediating role.

## INTRODUCTION

Youth represents a transitional period from childhood to adulthood characterized by significant physical, psychological, and social changes. During this period, behaviors with long-term health implications are formed, and individuals become increasingly vulnerable to adverse outcomes, particularly in the context of sexual health^1,2^. Sexuality is a natural component of human development; however, in many societies it remains a sensitive topic that is often surrounded by social taboos and limited communication within families. As a result, young people may obtain information from unreliable sources, which can lead to important knowledge gaps regarding sexual and reproductive health^2,3^. Insufficient knowledge may increase the risk of sexually transmitted diseases and other health problems^4,5^. The World Health Organization (WHO) reports that more than one million curable sexually transmitted infections are acquired every day worldwide among individuals aged 15–49, most of which are asymptomatic, and that university youth are often neglected within reproductive health programs^6^.

This lack of awareness and neglect may increase the likelihood of adverse health outcomes related to sexual contact, including AIDS and other sexually transmitted infections, unintended pregnancies, abortion, and other complications^7^.

In a healthy sexual life, individuals should be able to consult a physician when faced with sexual health problems and receive the necessary counseling, treatment, or referrals from health professionals^2,4,5,6^. University students may be considered a risk group because factors such as early initiation of sexual intercourse, having multiple partners, certain sexual preferences, relationships with commercial sex workers or their partners, not using condoms, being unmarried and young, living in large cities, and certain sociodemographic characteristics are associated with a higher risk of sexually transmitted diseases (STDs)^7^. Given the limited existing literature^2,3,7^, this study was designed not only to assess the current status of university students in Muğla province but also to examine, within the context of sexual and reproductive health, the role of knowledge in shaping appropriate health behaviors, as well as to observe the presence of misconceptions and the persistence of related negative attitudes.

## METHODS

### Study design and participants

This cross-sectional descriptive study was conducted between 14 September 2023 and 29 January 2024 among university students aged 18–26 years who applied to the Muğla Sıtkı Koçman University Kötekli No. 4 Family Health Center for any reason. A total of 383 volunteer students meeting the inclusion criteria were consecutively recruited during the study period.

### Data collection

The questionnaire was initially developed based on a literature review and was further refined by incorporating the opinions of specialists and faculty members from the gynecology clinic; it was subsequently reviewed by the Department of Family Medicine, after which the ethical approval process was initiated. A structured questionnaire consisting of 44 items was administered face-to-face; (1) sociodemographic characteristics (10 questions), (2) sexual health and family planning (18 questions), and (3) sexually transmitted diseases (16 questions). Participation was voluntary, and informed consent was obtained from all participants prior to the survey. “Sexual experience” was defined as engagement in penetrative sexual intercourse (vaginal or anal) to ensure consistency in interpretation among participants. Some questions allowed multiple responses; therefore, percentages may exceed 100%.

### Ethical approval

This study was reviewed and approved by the Medical Sciences Ethics Committee of Muğla Sıtkı Koçman University Faculty of Medicine with decision number 90 dated 28/09/2023 and protocol number 230087. The study was conducted in accordance with the Declaration of Helsinki and in compliance with the ethical standards of our country.

### Statistical analysis

Statistical analyses were performed using IBM SPSS Statistics version 26.0. Descriptive statistics were presented as frequencies and percentages. Associations between categorical variables were evaluated using the chi-square test and Fisher’s exact test, and a p<0.05 was considered statistically significant.

### Inclusion criteria

*Being 18–26 years of age

*Being a university student

*Voluntarily agreeing to participate in the study

No additional exclusion criteria were applied, and all participants were allowed to respond to all questionnaire items regardless of sexual experience.

## RESULTS

The mean age of the participants was 20.6±2.1 years (range: 18–26). Among sexually active participants, the mean age at first sexual intercourse was 18.9±2.0 years. Overall, 42.3% (n=162) reported having sexual experience, which was significantly more frequent among males than females (50.9 vs. 35.4%, p=0.002). Sexual experience was less common among medical students, with 70.7% (n=135) reporting no sexual experience (p<0.001). The internet was the most common source of sexual and reproductive health information (65.3%, n=250), whereas family was the least common (10.2%, n=39). Participants with sexual experience were more likely to obtain information from physicians compared to those without sexual experience (30.2 vs. 17.6%, p=0.004). Most participants indicated that sexual health education should be provided during high school (33.4%, n=128), followed by Family Health Centers (18.2%, n=70). Overall, participants demonstrated a high level of awareness regarding STDs, with human ımmunodeficiency virus/acquired ımmunodeficiency syndrome (HIV/AIDS) (n=293), gonorrhea (n=113), and human papillomavirus (HPV) (n=102) being the most frequently reported. Commonly identified symptoms included genital discharge (n=74), itching (n=55), and genital warts (n=41). Most participants reported no prior STD history (89.8%, n=344).

Although 95% (n=364) correctly identified condom use as the most effective method for STD prevention, misconceptions persisted. Notably, a considerable proportion of participants were unaware that STDs could cause cancer (40.4%, n=155), and those without sexual experience were more likely to lack knowledge about the protective role of condoms (p<0.001). Gender-based differences were evident in both knowledge and attitudes. Women were more likely to associate safe sexual behavior with monogamy (p<0.001) and pregnancy prevention (p=0.023), and to recognize the increased risk associated with multiple sexual partners (p=0.002). They were also more likely to endorse preventive strategies such as having a single partner (p=0.006) and avoiding contact with potentially infected individuals (p=0.001). In contrast, men were more likely to perceive higher STD risk among LGBTQ+ individuals (p=0.004). Condoms were the most commonly known contraceptive method, followed by oral contraceptive pills, emergency contraception, tubal ligation, and intrauterine devices, whereas the lactational method and diaphragm were the least known. Women demonstrated significantly higher knowledge of several contraceptive methods (p<0.05). While students without sexual experience had greater knowledge of oral contraceptive pills (p=0.045), knowledge of withdrawal (p=0.003) and emergency contraception (p<0.001) was higher among sexually experienced participants. Medical students showed higher knowledge of subdermal implants (p=0.011) and vasectomy (p=0.018), but lower knowledge of emergency contraception (p=0.002) compared to students from other faculties. The remaining findings are presented in Tables 1 and 2.

**Table 1:** Participants’ knowledge of transmission routes and risk groups of sexually transmitted infections.

Transmission routes of sexually transmitted infections according to sexual experience			
	N (F/M)	% (F/M)	p
Sexually transmitted diseases are transmitted only through sexual intercourse.			
No	311 (178/133)	81.2 (57.2/42.8)	0.700
Yes	72 (34/38)	18.8 (47.2/52.8)	
Sexually transmitted diseases can also be transmitted through blood or body fluids apart from sexual intercourse.			
No	55 (27/28)	14.4 (49.1/50.9)	0.709
Yes	328 (185/143)	85.6 (56.4/43.6)	
Sexually transmitted diseases are not transmitted through skin contact such as handshaking or kissing.			
No	278 (148/130)	72.6 (53.2/46.8)	0.892
Yes	105 (64/41)	27.4 (61/39)	
Some sexually transmitted diseases can be transmitted from mother to baby (during pregnancy, childbirth, or breastfeeding). p=0.035^*^			
No	161 (79/82)	42.0 (49.1/50.9)	0.137
Yes	222 (133/89)	58.0 (59.9/40.1)	
Sexually transmitted diseases cannot be transmitted through shared personal items (e.g., razors).			
No	328 (181/147)	85.6 (55.2/44.8)	0.709
Yes	55 (31/24)	14.4 (56.4/43.2)	
Perceived high-risk groups for sexually transmitted infections by gender			
The risk of sexually transmitted diseases is higher in women and men with multiple sexual partners.			
No	55 (20/35)	14.4 (36.4/63.6)	0.02^**^
Yes	328 (192/136)	85.6 (58.5/41.5)	
The risk of sexually transmitted diseases is higher among drug users.			
No	306 (171/135)	79.9 (55.9/44.1)	0.678
Yes	77 (41/36)	20.1 (53.2/46.8)	
The risk of sexually transmitted diseases is higher among individuals with a single sexual partner.			
No	377 (209/168)	98.4 (55.4/44.6)	0.790
Yes	6 (3/3)	1.6 (50/50)	
The risk of sexually transmitted diseases is higher among individuals with an active sexual life.			
No	242 (132/110)	63.2 (54.5/45.5)	0.677
Yes	141 (80/61)	36.8 (56.7/43.3)	
The risk of sexually transmitted diseases is higher among sex workers and their clients.			
No	76 (41/35)	19.8 (53.9/46.1)	0.783
Yes	307 (171/136)	80.2 (55.7/44.3)	
The risk of sexually transmitted diseases is higher among lesbian, gay, bisexual, and transgender individuals.			
No	226 (139/87)	59.0 (61.5/38.5)	0.004^*^
Yes	157 (73/84)	41.0 (46.5/53.5)	

*Fisher-exact test.

** Chi-square test.

**Table 2:** Transmission routes of sexually transmitted infections according to gender.

What are the methods for preventing sexually transmitted diseases?			
	N (F/M)	% (F/M)	p
Sexually transmitted diseases can be prevented by abstaining from sexual intercourse until marriage.			
No	343 (194/149)	89.6 (56.6/43.4)	0.181
Yes	40 (18/22)	10.4 (45/55)	
Sexually transmitted diseases can be prevented by using a condom during every sexual intercourse.			
No	91 (53/38)	23.8 (58.2741.8)	0.548
Yes	292 (159/133)	76.2 (54.5/45.5)	
Sexually transmitted diseases can be prevented by having a single sexual partner.			
No	150 (70/80)	39.2 (46.7/53.3)	0.06^**^
Yes	233 (142/91)	60.8 (60.9/39.1)	
Sexually transmitted diseases can be prevented by using antibiotics or similar medications.			
No	381 (212/169)	99.5 (55.6/44.4)	0.199
Yes	2 (0/2)	0.5 (0/100)	
Sexually transmitted diseases can be prevented by using oral contraceptive pills.			
No	345 (190/155)	90.1 (55.1/44.9)	0.740
Yes	38 (22/16)	9.9 (57.9/42.1)	
Sexually transmitted diseases can be prevented by avoiding sexual contact with individuals who may carry infectious diseases.			
No	75 (28/47)	19.6 (37.3/62.7)	0.001^*^
Yes	308 (184/124)	80.4 (59.7/40.3)	

*Fisher-exact test.

** Chi-square test.

Regarding abortion, men were more likely to agree that it should never be performed (p=0.032), whereas women were more likely to consider it a solution for unintended pregnancies (p=0.001). Students without sexual experience were significantly more likely to disagree with abortion as a solution (p<0.001) Table 3.

**Table 3:** Opinions about abortion.

What are your opinions about abortion?						
Gender				According to sexual experience		
	N (F/M)	% (F/M)	p	N (absent/present)	% (absent/present)	p
Abortion can be used to terminate unintended pregnancies.						
No	145 (69/76)	37.9 (40.4/35.8)	0.397	100/45	69/31	0.000^**^
Yes	238 (102/136)	62.1 (64.2/59.6)		121/117	50.8/49.2	
Abortion should be performed if medically necessary.						
No	154 (84/70)	40.2 (33.1/49.1)	0.002^*^	83/71	53.9/46.1	0.216
Yes	229 (87/142)	59.8 (50.9/67.0)		138/91	60.3/39.7	
Abortion should never be performed.						
No	364 (158/206)	95.0 (92.4/97.2)	0.056	210/154	57.7/42.3	0.986
Yes	19 (13/6)	5.0 (7.6/2.8)		11/8	57.9/42.1	
Abortion I have no opinion about it.						
No	351 (154/197)	91.6 (90.1/92.9)	0.355	199/152	56.7/43.3	0.186
Yes	32 (17/15)	8.4 (44.6/55.4)		22/10	68.8/31.3	

*Fisher-exact test.

**Chi-square test.

## DISCUSSION

The present study revealed three key findings. First, a relatively adequate level of sexual health knowledge was observed. Second, persistent misconceptions and stigmatizing attitudes coexisted with accurate knowledge. Third, knowledge did not consistently translate into appropriate health-related behaviors.

Sexuality is a natural part of the developmental process; however, initiating sexual intercourse before the age of 16—particularly sexual activity occurring at or before the age of 14—is considered a very early period in terms of physiological development^8^. The British National Survey of Sexual Attitudes and Lifestyles (Natsal-3) reported that 30.9% of individuals aged 16–24 had their first sexual intercourse before the age of 16, while the international Health Behaviour in School-aged Children report indicated that the age at first sexual intercourse in many European countries is approximately 15^8^. In our study, the observed age at first sexual experience ranging from 15 to 26 years and the rates varying between 16 and 36.4% demonstrate that early sexual experience is a significant public health issue on a global scale^7,9,10,11,12^.

From a human rights perspective, comprehensive sexuality education is considered a fundamental necessity^13^. The Sexuality Information and Education Council of the United States emphasizes that schools and families play a crucial role in meeting young people’s informational needs regarding human sexuality^13,14^. Indeed, sexuality education programs implemented in some countries have been shown to contribute positively to young people’s knowledge and awareness levels^1,2,13^. In this context, our study suggests that the significantly lower prevalence of sexual experience among medical students may be associated with the role of health literacy in shaping behavior and awareness. Additionally, the finding that individuals with sexual experience were more likely to obtain information from physicians may indicate a tendency to prefer more accurate and professional sources of information rather than passive sources, and may also reflect a shift toward more responsible health-seeking behavior. Furthermore, by demonstrating that a large proportion of participants believe sexual health education should be provided during high school, our findings suggest that young people are aware of their knowledge gaps and have a clear need for structured education in this area. Access to accurate information and comprehensive sexuality education plays a critical role in the development of healthy and safe sexual behaviors; however, some studies have shown that although awareness of sexually transmitted infections is widespread, significant misconceptions and false beliefs regarding modes of transmission and prevention methods persist^10,15,16,17^. In our study as well, alongside correct knowledge regarding HIV transmission, beliefs such as transmission through handshaking or kissing, and the perception that LGBTQ+ individuals are at higher risk, indicate a clear inconsistency between knowledge and attitudes, and demonstrate that accurate knowledge may coexist with misconceptions and stigmatizing attitudes. Additionally, since a multiple-response format was used in our study—allowing participants to select more than one option—some individuals may have simultaneously selected both correct and incorrect methods. We therefore consider that this may help explain the coexistence of accurate knowledge and misconceptions observed in our findings. This finding further supports the notion that having knowledge about sexually transmitted infections does not necessarily translate into correct and safe behaviors^10,17^. Although the majority of participants in our study were aware that condoms are protective, 22.7% reported not using a condom during their last sexual intercourse. Similarly, such inconsistencies, also reported in other studies^7,18,19^, reveal the discrepancy between knowledge and behavior, suggesting that awareness has not been fully achieved and that knowledge does not effectively translate into behavior^10,18,20,21^. This situation has been associated with individual and social factors such as trust in partners, difficulties in negotiating condom use, fear of social stigma, and a greater focus on preventing pregnancy rather than sexually transmitted infections^20^. These findings highlight the importance of addressing not only knowledge deficits but also underlying social and cognitive determinants of behavior.

In our study, despite men having higher levels of sexual experience and greater knowledge of emergency contraception, their tendency to view abortion as an option—without prioritizing preventive behaviors—reflects an approach that shifts responsibility away from themselves and resolves outcomes through women’s bodies^4,22^. This pattern underscores underlying gender norms in which responsibility for reproductive health is implicitly assigned to women, and suggests that women’s agency is often marginalized in sexual health decision-making processes.

In conclusion, although university students have knowledge regarding sexual and reproductive health, they do not always exhibit appropriate attitudes and behaviors. The coexistence of correct knowledge and persistent misconceptions indicates a significant gap between knowledge, attitudes, and behaviors. In this context, sexual and reproductive health requires a holistic approach that should be addressed from the perspectives of gender norms, responsibility, non-maleficence, and human rights.

### Limitations

This study has several limitations. First, it was conducted among students who applied to a single Family Health Center; therefore, the generalizability of the findings is limited. Second, the data were based on self-reported responses, which may have introduced reporting bias due to the sensitive nature of sexual behaviors. Third, due to the cross-sectional design, causal relationships cannot be established. Additionally, although the study aimed to assess risky sexual behaviors, behavioral measures were limited and the findings predominantly reflect knowledge and attitudes. Furthermore, participants were recruited from a healthcare setting, which may not fully represent the general student population. Additionally, we clarified this definition in the questionnaire description and acknowledged in the Limitations section that earlier interpretations of the term may have varied among participants, potentially introducing bias. Future multicenter studies involving larger populations are needed to better understand sexual health knowledge and behaviors among university students.

## Data Availability

The datasets generated and/or analyzed during the current study are available from the corresponding author upon reasonable request.
